# Anthropometric Predictors of Heart Rate Variability in Overweight Individuals: A Comparative Study

**DOI:** 10.7759/cureus.69434

**Published:** 2024-09-15

**Authors:** Nisha Surana Gandhi, Smita R Sorte, Dipali K Chatur, Sachin B Rathod

**Affiliations:** 1 Physiology, Dr. Vithalrao Vikhe Patil Foundation's Medical College, Ahmednagar, Ahmednagar, IND; 2 Physiology, All India Institute of Medical Sciences, Nagpur, Nagpur, IND; 3 Physiology, All India Institute of Medical Sciences, Raipur, Raipur, IND

**Keywords:** anthropometric parameters, autonomic nervous system dysfunction, autonomic nervous system imbalance, body mass index: bmi, heart rate variability (hrv), obesity, percentage body fat, sympathetic hyperactivity, waist circumference, waist-hip ratio

## Abstract

Introduction

The autonomic nervous system is crucial in regulating cardiovascular function. Heart rate variability (HRV) analysis, a non-invasive method to assess autonomic function, reflects the beat-to-beat variations in heart rate and provides insights into the dynamic interplay between sympathetic and parasympathetic influences on the cardiovascular system. In this study, we aimed to find the HRV parameters in overweight individuals by comparing different anthropometric parameters, including body mass index (BMI), percentage body fat (%BF), waist circumference (WC), and waist-hip ratio (WHR).

Method

The descriptive cross-sectional study was conducted on 132 healthy first-year MBBS students (82 males and 50 females), aged between 17 and 23 years. Anthropometric parameters (BMI, %BF, WC, WHR) and HRV were recorded for the participants. The HRV parameters were analyzed for either gender. A student 't' test was used to test the difference between groups BMI <25 kg/m^2^ and = or >25 kg/m^2^, and a p-value less than 0.05 was taken to be statistically significant. The Pearson correlation coefficient was used to show the relationship between HRV parameters and BMI, %BF, WC, and WHR as the independent variables.

Result

A total of 132 healthy individuals participated in the study, including 82 males and 50 females. The participants had a mean age of 18.72 ± 0.98 years. Our results suggest that while WC and WHR can be used alongside BMI to indicate sympathetic hyperactivity in males, BMI remains the most significant predictor in females. We found gender-specific differences in high-frequency (HF) related to various anthropometric measures, but these differences were not statistically significant. The low-frequency:high-frequency (LF/HF) ratio exhibits strong positive correlations with all measured anthropometric parameters, suggesting that increased values in BMI, %BF, WC, and WHR are associated with greater sympathetic involvement.

Conclusion

WC and WHR, alongside BMI, are reliable indicators of heightened sympathetic activity in both males and females. These measures should be utilized to assess healthy individuals for the early diagnosis of dysautonomia, disrupt the cycle of sympathetic overactivity, and prevent cardiovascular complications. All these anthropometric parameters are valuable for indicating sympathovagal balance, making them essential tools in the proactive management of autonomic dysfunction and associated health risks.

## Introduction

Background

Obesity is a condition characterized by excessive adipose tissue accumulation in the body. It is now understood that adipose tissue is not merely a passive energy storage site but a complex organ involved in various physiological processes [1]. The prevalence of obesity has surged to alarming levels worldwide, making it one of the most pressing public health challenges of the 21st century. According to the World Health Organization (WHO), the global prevalence of obesity has nearly tripled since 1975. The rise in obesity is driven by a complex interplay of factors, including sedentary lifestyles, increased consumption of high-calorie, nutrient-poor foods, genetic predispositions, and socioeconomic influences. The high prevalence of obesity is associated with a significant burden of comorbidities, such as type 2 diabetes, cardiovascular diseases, and certain cancers, leading to increased healthcare costs and reduced quality of life [2]. The pathophysiological mechanisms linking obesity and cardiovascular dysfunction are complex and multifaceted. Among these mechanisms, cardiovascular autonomic dysfunction has emerged as a significant contributor to the increased cardiovascular risk observed in obese individuals [3].

WHO-classified body mass index (BMI), also known as the Quetelet index, has been widely used to assess the relationship between body weight and height. However, BMI alone may not be sufficient to comprehensively understand an individual's health status. BMI has limitations as it does not differentiate between fat, muscle, and bone mass. Consequently, individuals with dense bones or well-developed muscles may be misclassified as overweight or obese, while individuals with a normal BMI ranging from 18.5-24.9 may still have a high percentage of body fat, which can be detrimental to their health.

To accurately assess the health implications of obesity, it is essential to consider other factors such as body composition, distribution of adipose tissue, and metabolic health markers. These considerations provide a more comprehensive evaluation of an individual's overall health and the associated risks related to obesity [4]. Visceral fat refers to the fat stored around the organs in the abdominal cavity that can be obtained by measuring waist circumference (WC). It helps identify individuals at a higher risk of developing obesity-related complications such as heart disease, stroke, type 2 diabetes, and some types of cancer [5].

Percentage body fat (%BF) provides a representation of an individual's body composition by distinguishing between lean mass (muscles, bones, organs, and other non-fat tissues) and fat mass. This precision is crucial for identifying individuals at risk of obesity-related health complications. Some individuals may have a normal BMI but a high %BF, a condition known as "normal-weight obesity," which %BF can help identify. %BF enhances the precision and comprehensiveness of health assessments, can better stratify risk, and tailor interventions to reduce these risks.

Waist-hip ratio (WHR), an anthropometric measurement, is the ratio of the circumference of the waist to the circumference of the hips. This ratio provides an indication of fat stored around their abdomen (visceral fat) compared to their hips (subcutaneous fat). A higher WHR indicates a greater concentration of visceral fat and central obesity. WHR is considered a more reliable indicator of fat distribution as it accounts for differences in body shape and fat storage patterns.

%BF, WC, and WHR are simple, non-invasive, and cost-effective measurements requiring minimal equipment and training that can be easily performed in clinical and research settings, making them a practical tool for large-scale health assessments.

The autonomic nervous system (ANS) plays a crucial role in regulating cardiovascular function, maintaining homeostasis, and adapting to physiological demands. Its two main branches, the sympathetic and parasympathetic systems, work harmoniously to modulate heart rate, blood pressure, and vascular tone [6]. However, in obesity, this delicate balance may be disrupted, leading to autonomic imbalance and dysfunction [7]. ANS dysfunction plays a dual role in the context of obesity. Firstly, ANS disturbances may contribute to the development of obesity, playing a role in its pathogenesis [7]. Conversely, excess weight can lead to ANS dysfunction [8], further exacerbating the hemodynamic and metabolic changes, thereby increasing the cardiovascular risk in obese individuals. These changes include conditions such as hypertension, insulin resistance, and dyslipidemia.

Heart rate variability (HRV) analysis, a non-invasive method to assess autonomic function, reflects the beat-to-beat variations in heart rate and provides insights into the dynamic interplay between sympathetic and parasympathetic influences on the cardiovascular system [9]. Alterations in HRV parameters have been observed in obese individuals, indicating impaired autonomic regulation.

Research gap

Despite extensive research on obesity and its related health risks, there is still a lack of comprehensive understanding regarding the specific impact of various anthropometric parameters on cardiovascular autonomic dysfunction. While BMI has been extensively studied, other measures such as %BF, WC, and WHR have not been thoroughly investigated in the context of their relationship with autonomic function. Moreover, the interaction between different types of fat distribution and autonomic imbalance remains underexplored, particularly in terms of their combined effect on cardiovascular health.

Bridging the gap

This study seeks to bridge the existing research gap by providing a detailed understanding of how different measures of body composition and fat distribution affect autonomic regulation in obese individuals. This approach will offer more nuanced insights into the interplay between obesity and autonomic dysfunction, highlighting the importance of considering multiple anthropometric measures for a comprehensive health assessment.

Aim of the study

The primary aim of this study is to provide a relationship between cardiovascular autonomic parameters with a specific focus on insights derived from HRV analysis and various anthropometric parameters, including BMI, %BF, WC, and WHR. Additionally, the study aims to highlight the potential clinical implications of HRV analysis in identifying and monitoring cardiovascular autonomic dysfunction in obese individuals. Ultimately, this research seeks to enhance our understanding of the complex interplay between obesity and autonomic dysfunction, paving the way for improved patient management and better outcomes in addressing obesity-related cardiovascular diseases.

## Materials and methods

The descriptive cross-sectional study was conducted in the Department of Physiology (Neurophysiology Research Laboratory) of Jawaharlal Nehru Medical College, Datta Meghe Institute of Medical Sciences University (Deemed University), Sawangi (Meghe), Wardha, after obtaining ethical clearance from the Institutional Ethical Committee (reference no. DMIMS(DU)/IEC/2010-11/75 dated 30.09.2010).

The study included 132 healthy first-year MBBS students of either sex (82 males and 50 females) and aged between 17 and 23 years. Informed consent was obtained from the students. Students who were not willing to participate or absent (12) were excluded from the study. The study duration was six months.

Students with a history of alcohol intake (15), smoking (32), hypertension (>140/90), diabetes (2), parental history of hypertension or diabetes (5), anemia, recent viral/bacterial infections (2), adrenal, thyroid disorders, cardiovascular, respiratory diseases, any drug intake, unstable body weight (change of >1% within the month before the study), or post-operative were excluded.

Students who fulfilled the inclusion criteria were invited to the Neurophysiology Research Laboratory, and anthropometric parameters like height, weight, BMI, WC, WHR, and %BF were recorded. Weight and height were measured with light clothes, without footwear and a coat.

The subject stood against a wall with their feet together, ensuring their heels, buttocks, and occiput (back of the head) firmly touched the wall. They maintained an upright head position, looking straight ahead without any tilting. The highest point on their head was marked on the wall with a plastic ruler to measure the height. The height was measured up to the nearest centimeter from this marked point.

The subjects' body weight was determined using a pedestal-style weighing scale capable of measuring up to a maximum of 150 kg. The body weight was recorded to the nearest 0.1 kg. The scale was placed on a hard, flat surface to prevent tilting or imbalance. It was turned on and calibrated to zero using the "zero" function. A known weight was placed on the scale to check accuracy. The weighing area was kept free from drafts, vibrations, and other environmental factors that could affect performance, and the scale was protected from extreme temperatures and humidity. This rigorous standardization ensured reliable and precise measurements throughout the study.

The BMI is computed by dividing an individual's weight by the square of their height: BMI = weight in kgs/(height in square meters) [6]. Waist and hip circumference were assessed using stretch-resistant tape. The tape was wrapped around the subject snugly but without causing any constriction. The measurement was taken with the tape kept level and parallel to the floor at the designated points. The subject stood upright, arms relaxed at their sides, feet evenly spaced apart, and body weight evenly distributed.

WC was measured at the highest points of the two iliac crests at a level parallel to the floor. The measurement was taken at the end of several consecutive natural breaths [6]. Hip circumference is determined by measuring the buttocks' largest circumference, ensuring the measurement level remains parallel to the floor.

The percentage of body fat was calculated by the Deurenberg method [7]. It is calculated by the equation: adult body fat % = (1.20 x BMI) + (0.23 x Age) - (10.8 x gender) -5.4 {where male gender=1, female=0}

The evaluation of ANS functions was done by HRV. The procedure was thoroughly explained to the participants. The influence of diurnal variations was minimized by conducting all tests between 10:00 am and 12:00 noon. Participants were instructed to abstain from consuming coffee, tea, and cola for 12 hours before the tests. They were also advised to have a light breakfast two hours before the tests. Upon arrival at the laboratory, the participant was asked to relax supine for 15 minutes.

The resting HRV assessment was performed using the computerized advanced RMS polyrite system from Chandigarh. This system digitally converted the analog data and provided software for data acquisition and analysis. The resting HRV was recorded for 132 participants to quantify the autonomic drive to the myocardium. Power spectral analysis of the resting electrocardiogram (ECG) signal was conducted using fast Fourier transformation (FFT). The ECG signal was first converted from analog to digital using an A/D converter with a sampling frequency of 256 Hz. Both frequency and time domain analyses were performed on the data.

FFT was applied using Welch's Periodogram Method with a Hann Window for the frequency domain analysis. Through the frequency domain analysis, several parameters were obtained using power spectral density analysis. These parameters included low-frequency (LF) power, high-frequency (HF) power, and the LF/HF ratio.

The LF component, ranging from 0.04 to 0.15 Hz, reflects the activity of baroreceptors, i.e., the sympathetic drive to the myocardium. On the other hand, the HF component, ranging from 0.15 to 0.4 Hz, represents parasympathetic activity. The LF/HF ratio indicates the balance between sympathetic and parasympathetic regulation, commonly called sympathovagal balance [8]. To compute HRV indices during supine rest, the recommendations of the Task Force on HRV were followed [9].

Statistical analysis

Descriptive statistics were applied. The results were averaged by the mean ± standard deviation for each anthropometrical parameter for males and females. A student 't' test was used to test the difference between groups BMI <25 kg/m^2^ and = or >25 kg/m^2^, and a p-value less than 0.05 was taken to be statistically significant. The normality of data distribution was tested for all the parameters and was found to be normally distributed. The Pearson correlation coefficient was used to show the relationship between HRV parameters and BMI, %BF, WC, and WHR as the independent variables. The data were analyzed using the statistical software Jamovi 2.8.28 solid version (Jamovi, Sydney, Australia, 2024) [10].

## Results

A total of 132 healthy students participated in the study, consisting of 82 males and 50 females. The participants had a mean age of 18.72 ± 0.98 years. The mean BMI was 23.19 ± 2.96 for all participants, with no significant difference between males (23.19 ± 3.29) and females (23.18 ± 2.37) (p=0.982). The mean %BF was significantly higher in females (26.72 ± 2.86) compared to males (15.94 ± 3.96), with a p-value of <0.001, indicating a statistically significant difference. The average WC was significantly higher in males (84.26 ± 4.02 cm) than in females (72.70 ± 6.10 cm), with a p-value of <0.001. The mean WHR was significantly higher in males (0.815 ± 0.05) compared to females (0.78 ± 0.04), with a p-value of 0.001. These results highlight significant gender differences in %BF, WC, and WHR, while BMI did not show a significant difference between genders (Table 1).

**Table 1:** Participants' mean and SD for anthropometrical parameters BMI: body mass index, %BF: percentage body fat, WC: waist circumference, WHR: waist-hip ratio, SD: standard deviation

Anthropometrical parameter	All participants (n=132)		Males (n=82)		Females (n=50)		Unpaired T-test
	Mean	SD	Mean	SD	Mean	SD	p-value
BMI (kg/m^2^)	23.19	2.96	23.19	3.29	23.18	2.37	0.982
%BF (%)	20.02	6.35	15.94	3.96	26.72	2.86	<0.001
WC (cm)	79.88	7.45	84.26	4.02	72.7	6.10	<0.001
WHR	0.80	0.05	0.815	0.05	0.78	0.04	0.001

LF component compared with anthropometric parameters in males

The LF component of HRV was significantly higher in males with a BMI ≥25 (318.96 ± 234.20) compared to those with a BMI <25 (171.26 ± 202.08) (p<0.01). Similarly, LF was significantly elevated in males with a WC ≥90 cm (436.61 ± 203.19) compared to those with a WC <90 cm (179.46 ± 205.15, p<0.01). LF was also significantly higher in males with a WHR (WHR) ≥0.9 (436.61 ± 203.19) compared to those with a WHR <0.9 (179.46 ± 205.15, p<0.01). No significant difference in LF was observed to %BF (p>0.05).

HF component compared with anthropometric parameters in males

The HF component of HRV showed no significant variation in BMI (p>0.05), %BF (p>0.05), or WC (p>0.05). However, HF was higher in males with a BMI ≥25 (121.40 ± 136.77) compared to those with a BMI <25 (104.43 ± 103.18), although this difference was not statistically significant. Similarly, HF was lower in males with %BF ≥25 (88.98 ± 15.53) compared to those with %BF <25 (107.99 ± 110.64), with no significant difference (p>0.05). HF also showed no significant difference to WHR (p>0.05).

LF/HF component compared with anthropometric parameters in males

The LF/HF ratio, indicative of balance between sympathetic and parasympathetic activity, was significantly higher in males with a BMI ≥25 (3.32 ± 1.00) compared to those with a BMI <25 (1.55 ± 0.50, p<0.01). The LF/HF ratio was also significantly higher in males with a WC ≥90 cm (3.88 ± 1.06) compared to those with a WC <90 cm (1.72 ± 0.71, p<0.01). Similarly, males with a WHR ≥0.9 had a significantly higher LF/HF ratio (3.88 ± 1.06) compared to those with a WHR <0.9 (1.72 ± 0.71, p<0.01). Significant difference in the LF/HF ratio was observed to %BF (p<0.01).

The analysis reveals that in males, the LF and LF/HF ratios show significant differences across BMI, WC, and WHR categories, indicating that these parameters are sensitive markers for assessing the impact of anthropometrical parameters on HRV. HF, on the other hand, does not show significant differences in most comparisons (Table 2).

**Table 2:** Comparison of HRV spectral analysis with different anthropometrical parameters in males Group comparison is done by paired T-test HRV: heart rate variability, LF: low-frequency power, HF: high-frequency power, BMI: body mass index, %BF: percentage body fat, WC: waist circumference, WHR: waist-hip ratio, SD: standard deviation

Anthropometrical parameter (males)		LF (ms^2^) paired T-test			HF (ms^2^) paired T-test			LF/HF paired T-test		
		Mean	SD	P	Mean	SD	P	Mean	SD	P
BMI (kg/m^2^)	<25	171.2591	202.0792	<0.01	104.4276	103.1757	>0.05	1.551045	0.497129	<0.01
	≥25	318.9647	234.2049		121.4047	136.7712		3.322067	1.004031	
%BF (%)	<25	196.3135	216.9257	>0.05	107.997	110.6402	>0.05	1.831763	0.877769	<0.01
	≥25	276.875	18.56155		88.98	15.52806		3.605	1.364716	
WC (cm)	<90	179.4632	205.1459	<0.01	105.1713	110.4956	>0.05	1.716842	0.705355	<0.01
	≥90	436.605	203.1886		137.45	137.45		3.8785	1.055085	
WHR	<0.9	179.4632	205.1459	<0.01	105.1713	110.4956	>0.05	1.716842	0.705355	<0.01
	≥0.9	436.605	203.1886		137.45	96.45303		3.8785	1.055085	

LF component compared with anthropometric parameters in females

The LF component was significantly higher in females with a BMI ≥25 (438.414 ± 323.423) compared to those with a BMI <25 (201.776 ± 235.366), with a p-value of <0.01. There was no significant difference in the LF component between females with %BF ≥25 (217.06 ± 241.583) and those with %BF <25 (259.22 ± 279.56), with a p-value of >0.05. The LF component showed no significant difference between females with a WC ≥80 cm (254.64 ± 98.514) and those with a WC <80 cm (248.488 ± 282.477), with a p-value of >0.05. The LF component was not significantly different between females with a WHR ≥0.85 (254.64 ± 111.441) and those with a WHR <0.85 (248.488 ± 279.387), with a p-value of >0.05.

HF component compared with anthropometric parameters in females

The HF component did not show a significant difference between females with a BMI ≥25 (167.961 ± 147.832) and those with a BMI <25 (113.546 ± 114.227), with a p-value of >0.05. There was no significant difference in the HF component between females with %BF ≥25 (126.92 ± 141.79) and those with %BF <25 (123.64 ± 117.173), with a p-value of >0.05. The HF component showed no significant difference between females with a WC ≥80 cm (69.106 ± 24.701) and those with a WC <80 cm (130.576 ± 127.011), with a p-value of >0.05. The HF component was not significantly different between females with a WHR ≥0.85 (68.887 ± 27.939) and those with a WHR <0.85 (139.943 ± 125.831), with a p-value of >0.05.

LF/HF component compared with anthropometric parameters in females

The LF/HF ratio was significantly higher in females with a BMI ≥25 (3.146 ± 0.966) compared to those with a BMI <25 (1.699 ± 0.584), with a p-value of <0.01. There was no significant difference in the LF/HF ratio between females with %BF ≥25 (1.69 ± 0.656) and those with %BF <25 (2.08 ± 0.935), with a p-value of >0.05. The LF/HF ratio was significantly higher in females with a WC ≥80 cm (3.978 ± 0.561) compared to those with a WC <80 cm (1.767 ± 0.586), with a p-value of <0.01. The LF/HF ratio was significantly higher in females with a WHR ≥0.85 (3.83 ± 0.523) compared to those with a WHR <0.85 (1.83 ± 0.713), with a p-value of <0.01.

The analysis reveals that in females, the LF/HF ratios show significant differences across BMI, WC, and WHR, indicating that these parameters are sensitive markers for assessing the impact of anthropometrical parameters on HRV. Higher BMI, larger WC, and higher WHR are all associated with increased sympathetic dominance. Conversely, the %BF does not show a significant influence on the LF, HF, or LF/HF ratio, suggesting it may not be a primary determinant of the autonomic balance between sympathetic and parasympathetic activity. HF, on the other hand, does not show significant differences with any of the anthropometric parameters (Table 3).

**Table 3:** Comparison of spectral analysis concerning different anthropometrical parameters in females Group comparison is done by paired T-test LF: low-frequency power, HF: high-frequency power, BMI: body mass index, %BF: percentage body fat, WC: waist circumference, WHR: waist-hip ratio, SD: standard deviation

Anthropometrical parameter (females)	Groups	LF (ms^2^) paired T-test			HF (ms^2^) paired T-test			LF/HF paired T-test		
		Mean	SD	P	Mean	SD	P	mean	SD	P
BMI (kg/m^2^)	<25	201.776	235.3656	<0.01	113.546	114.2272	>0.05	1.699	0.583688	<0.01
	≥25	438.414	323.423		167.961	147.8321		3.146	0.966221	
%BF (%)	<25	259.22	279.560	>0.05	123.64	117.173	>0.05	2.08	0.935	>0.05
	≥25	217.06	241.583		126.92	141.790		1.69	0.656	
WC (cm)	<80	248.4884	282.4766	>0.05	130.576	127.011	>0.05	1.767333	0.58757	<0.01
	≥80	254.64	98.51423		69.106	24.7011		3.978	0.560687	
WHR	<0.85	248.4884	279.387	>0.05	129.43	125.831	>0.05	1.83	0.713	<0.01
	≥0.85	254.64	111.441		66.88	27.939		3.83	0.523	

LF component Pearson's correlation with anthropometric parameters

The LF component, reflecting sympathetic activity, shows a significant positive correlation with BMI (r=0.252, p<0.05). This indicates that higher BMI is associated with increased sympathetic activity. Similarly, the %BF also shows a positive correlation with LF (r=0.258), though it is not statistically significant. WC demonstrates the strongest positive correlation with LF (r=0.359, p<0.001), suggesting that larger WC is significantly associated with increased sympathetic activity. The WHR also shows a positive correlation with LF (r=0.218), though this correlation is not statistically significant.

HF component Pearson's correlation with anthropometric parameters

The HF component, indicative of parasympathetic activity, does not show significant correlations with any of the anthropometric parameters. The correlation coefficients for BMI (r=0.073), %BF (r=0.086), WC (r=0.199), and WHR (r=0.092) are all positive but not statistically significant. This suggests that parasympathetic activity, as measured by HF, is not strongly influenced by these anthropometric measures.

LF/HF ratio Pearson's correlation with anthropometric parameters

The LF/HF ratio, representing the balance between sympathetic and parasympathetic activity, shows significant positive correlations with all the anthropometric parameters. BMI has a very strong positive correlation with the LF/HF ratio (r=0.617, p<0.001), indicating that higher BMI is associated with greater sympathetic dominance. Similarly, the %BF also shows a strong positive correlation with the LF/HF ratio (r=0.609, p<0.001), suggesting increased sympathetic dominance with a higher %BF. WC (r=0.474, p<0.001) and WHR (r=0.398, p<0.001) also show significant positive correlations with the LF/HF ratio, indicating that larger measurements in these parameters are associated with increased sympathetic dominance.

The LF component shows significant positive correlations with BMI and WC, indicating increased sympathetic activity with higher values in these parameters. The HF component does not show significant correlations with any anthropometric measures, suggesting that parasympathetic activity is not strongly influenced by these parameters. The LF/HF ratio exhibits strong positive correlations with all measured anthropometric parameters, suggesting that increased values in BMI, %BF, WC, and WHR are associated with greater sympathetic dominance (Table 4).

**Table 4:** Pearson's correlation between the parameters of spectral analysis and different anthropometrical parameters for all participants LF: low-frequency power, HF: high-frequency power, BMI: body mass index, %BF: percentage body fat, WC: waist circumference, WHR: waist-hip ratio Note. * p<0.05, *** p<0.001

Anthropometrical parameters (all participants)	LF r-value	HF r-value	LF/HF r-value
BMI (Kg/m^2^)	0.252*	0.073	0.617***
%BF (%)	0.258	0.086	0.609***
WC (cm)	0.359***	0.199	0.474***
WHR	0.218	0.092	0.398***

## Discussion

The ANS consists of two branches with opposing actions: the sympathetic nervous system (SNS) and the parasympathetic nervous system. These systems work in a balanced manner, inhibiting each other's activity. The interplay between the sympathetic and parasympathetic branches is reflected in HRV, which measures the interval variations between heartbeats. Chronic alterations of the ANS will significantly influence the functions of the cardiovascular system [11]. There is a notable correlation between ANS regulation and mortality related to cardiovascular diseases [12]. HRV has been identified as a predictor of post-myocardial infarction mortality [13]. Hence, HRV is helpful in screening and preventing the development of life-threatening cardiovascular diseases [14]. Maintaining a balanced and healthy ANS function is essential for optimal cardiovascular function and longevity. In this study, we aimed to find the HRV parameters in overweight individuals by comparing different anthropometric parameters: BMI, %BF, WC, and WHR.

LF component in overweight and obese males and females as compared with different anthropometric parameters

The LF component reflects the activity of baroreceptors, i.e., the sympathetic drive to the myocardium. The analysis of LF components concerning various anthropometric indices reveals critical insights into gender-specific patterns of sympathetic hyperactivity.

Our study demonstrated that LF was statistically higher in subjects with a BMI greater than 25 kg/m² for both males and females, underscoring the relationship between elevated BMI and increased SNS activity across genders. This aligns with the understanding that higher BMI, indicative of overweight and obesity, correlates with the heightened sympathetic drive, which is driven by higher levels of pro-inflammatory adipokines and fatty acids, impaired arterial baroreceptor function, insulin resistance, and increased circulating leptin and leptin receptor signaling in obesity [15].

Straznicky et al. corroborated our findings, indicating that obesity is strongly correlated with an elevation in sympathetic nerve activity [16]. WC is a crucial indicator of central adiposity and is often linked to cardiovascular risk [17]. WC provided another dimension of insight, with LF being higher in males with a WC greater than 90 cm and in females with a WC greater than 80 cm. However, statistical significance was found only in males. This indicates that central adiposity, as reflected by WC, is a more reliable indicator of sympathetic hyperactivity in males than in females [18].

WHR is an important marker for assessing the risk of metabolic and cardiovascular diseases. A higher WHR in males indicates a more significant central fat accumulation, which correlates with a higher risk of metabolic syndrome and cardiovascular complications [16]. Similarly, the WHR revealed that LF was higher in males with a WHR greater than 0.9 and in females with a WHR greater than 0.85, but statistical significance was found only in males. This further supports the notion that WHR, a marker of fat distribution, is a significant indicator of sympathetic activity in males but not in females.

Guarino et al. proposed that changes in sympathetic and parasympathetic nerve activity, selective leptin resistance, low ghrelin levels, and hyperinsulinemia are possible mechanisms underlying sympathetic activation in obesity [19]. Selective leptin resistance, decreased levels of ghrelin (a hormone associated with hunger), and elevated levels of insulin collectively may play a role in driving the sympathetic activation observed in individuals with obesity [20]. Leptin is a hormone produced by adipose tissue. Elevated levels of leptin often characterize obesity due to increased fat mass. While leptin is primarily known for its role in appetite regulation and energy expenditure, it can also affect SNS activity. Studies have suggested that leptin may have direct sympathetic stimulatory effects [21]. Insulin resistance, a condition commonly associated with obesity, occurs when cells become less responsive to the effects of insulin. Insulin resistance can lead to increased sympathetic nerve activity. Studies have shown that insulin resistance positively correlates with sympathetic overactivity in obese individuals [22].

Overall, these findings highlight that WC and WHR, alongside BMI, are good indicators of sympathetic hyperactivity in males. Conversely, in females, BMI is a more reliable indicator of sympathetic activity than other anthropometric measures. This gender-specific distinction is crucial for developing tailored interventions to manage sympathetic hyperactivity and associated health risks.

HF component in overweight males and females as compared with different anthropometric parameters

The HF component, indicative of parasympathetic activity, does not show significant correlations with any of the anthropometric parameters. This highlights the complexity of the relationship between body composition and autonomic function and suggests that other factors may play a more substantial role in influencing HF. Molfino et al. got an inverse relationship between BMI and HF [23]. The studies by Arrone et al. observed significant weight gain accompanied by reduced parasympathetic activity in non-obese subjects. Conversely, substantial weight loss was linked to decreased sympathetic activity and increased parasympathetic activity [24]. Similarly, Westphale et al. also reported an association between sympathovagal balance and BMI in non-obese, healthy individuals [25].

LF/HF component in overweight males and females as compared with different anthropometric parameters

The LF/HF ratio indicates the balance between sympathetic and parasympathetic regulation, commonly called sympathovagal balance [7]. The LF/HF ratio exhibits strong positive correlations with all measured anthropometric parameters, suggesting that increased values in BMI, %BF, WC, and WHR are associated with greater sympathetic dominance. However, the gender differences observed concerning %BF suggest that there may be underlying physiological or hormonal factors influencing these patterns, particularly in females.

Obesity is characterized by chronic low-grade inflammation. Adipocytes, or fat cells, can release vasoactive hormones and factors that can influence metabolism and cardiovascular regulation. Among these substances are tumor necrosis factor α, interleukin 6, and leptin. Tumor necrosis factor α is a cytokine released by adipocytes that can harm metabolism and cardiovascular health. It is associated with insulin resistance, inflammation, and the development of cardiovascular diseases. Interleukin 6 (IL-6), another cytokine produced by adipocytes, has anti-inflammatory effects in some situations; elevated levels of IL-6 in obesity can contribute to chronic low-grade inflammation and insulin resistance, further impacting metabolic and cardiovascular functions. In obesity, the body can resist leptin's effects, leading to an imbalance in appetite control and potential disruptions in metabolism and cardiovascular regulation.

The release of these vasoactive hormones and factors by adipocytes underscores the intricate connection between adipose tissue, metabolism, and cardiovascular health. Their effects on various physiological processes highlight the importance of understanding adipose tissue function and its implications for overall well-being. These substances can impact metabolism and cardiovascular regulation [26]. Additionally, adipose tissue is closely linked to the development of obesity, diabetes, and various other complications [27].

Dysfunctional adipose tissue in obesity can disrupt normal adipokine secretion. Adipokines mainly involved in obesity and MetS are leptin, nonesterified free fatty acids, reactive oxygen species, adipocytic angiotensinogen, and resistin. Chronic dysregulation of certain adipokines can have deleterious effects on insulin signaling. Altered adipokine production can contribute to sympathetic overactivity [28].

In obesity, the renin-angiotension-aldesterone system (RAAS) may be upregulated; plasma renin activity is increased, which regulates blood pressure and fluid balance. Activation of the RAAS can increase sympathetic activity [29].

Animal studies have revealed that angiotensin II, a hormone that regulates blood pressure, can exert its effects centrally by enhancing SNS outflow. These findings suggest that angiotensin II may increase central SNS outflow in overweight or obese individuals [30]. Obesity is a common risk factor for obstructive sleep apnea, which can contribute to sympathetic overactivity through intermittent hypoxia and arousal during sleep. Psychological stress, depression, and anxiety, which are often associated with obesity, can activate sympathetic pathways and contribute to sympathetic overactivity.

The increased sympathetic activity observed with increased body fat may also be attributed to baroreflex impairment, including cardiac and peripheral control. This impairment reduces the inhibitory signals to the vasomotor center. It reduces the distensibility of the arterial wall, where these baroreceptors are situated, potentially a significant factor contributing to sympathetic activation in obesity [29].

Limitations

The study was conducted on a relatively small and homogeneous sample of first-year MBBS students, which limits the generalizability of the findings to the broader population. All participants were of a similar age, having just entered medical school, and shared a uniform educational background, as age and educational experiences can impact physiological responses and stress levels. Consequently, the results may not apply to older individuals or those with different educational or professional backgrounds or the general population's characteristics. While BMI, %BF, WC, and WHR were considered, other potentially relevant anthropometric or metabolic markers were not included, such as visceral fat measurement, lipid profiles, or inflammatory markers. These could provide a more comprehensive understanding of the relationship between obesity and autonomic function. Factors such as physical activity levels, diet, sleep patterns, and psychological stress were not controlled for or assessed in this study. These factors can significantly influence HRV and may confound the relationship between obesity and autonomic function. The exclusion of individuals with a history of smoking, alcohol intake, hypertension, or other medical conditions may have led to a sample that does not fully represent the diversity of the population, potentially limiting the external validity of the findings. As a cross-sectional study, the research only provides a snapshot of the relationship between anthropometric parameters and HRV at a single point in time. This design cannot establish causality or track changes over time, limiting the ability to observe long-term trends or effects.

## Conclusions

Being overweight and obese have become significant health issues and are more susceptible to developing chronic ailments with irreversible consequences. Early detection and intervention to reverse the changes in the body before the onset of irreversible diseases could substantially reduce morbidity rates in these individuals. Our study aligns with previous research findings that have consistently reported sympathetic overactivity in individuals with obesity. WC and WHR, in addition to BMI, are reliable indicators of heightened sympathetic activity in both males and females. These measures should be used to assess healthy individuals for early diagnosis of dysautonomia, disrupt the cycle of sympathetic overactivity, and prevent cardiovascular complications. Targeting the modulation of sympathetic activity may hold potential for therapeutic strategies aimed at managing body weight and mitigating the adverse effects of obesity on overall health.
